# The changing face of chemotherapy in colorectal cancer

**DOI:** 10.1054/bjoc.2000.1552

**Published:** 2001-01-01

**Authors:** J Waters, D Cunningham

**Affiliations:** Royal Marsden Hospital, London and Surrey, UK


*British Journal of Cancer* (2001) **84**(1), 1–7
© 2001 Cancer Research Campaign
doi: 10.1054/ bjoc.2000.1552, available online at http://www.idealibrary.com on IDEAL®

Review

### The changing face of chemotherapy in colorectal cancer

**J Waters and D Cunningham**

Royal Marsden Hospital, London and Surrey, UK

Cytotoxic chemotherapy for colorectal cancer has undergone a period of dramatic development over the course of the last 5 years. Four distinct classes of drug with activity in this disease are now available, and the current challenge is to establish the best way to use these agents, either in sequence or in combination, for the benefit of patients. This review aims to summarize the data relating to the newer agents and to propose directions for future research.

### DRUGS TARGETING THYMIDYLATE SYNTHASE

5-fluorouracil (5-FU) has established single-agent activity in advanced colorectal cancer. Its main intracellular target is thymidylate synthase, which is inhibited by the active metabolite of 5-FU, 5-fluorodeoxyuridine monophosphate (FdUMP) (Figure 1). The lack of alternative agents until recent years has fuelled extensive research into the biochemical modulation of 5-FU and alternative methods of delivery, primarily based on continuous infusion of the drug. Many individual clinical trials have demonstrated enhanced response rates when 5-FU is co-administered with folinic acid (calcium leucovorin). However a significant survival benefit has been more difficult to establish. Indeed a meta-analysis of nine trials showed no survival advantage for folinic acid-modulated 5-FU over 5-FU alone (Advanced Colorectal Cancer Meta-Analysis Project, 1992). Similarly, continuous infusion of 5-FU using a variety of different schedules also enhances response rates over bolus delivery. A meta-analysis of seven randomized trials addressing this question showed a small but statistically significant improvement in median survival for 5-FU infusion over 5-FU bolus (12.1 vs 11.3 months, $P = 0.039$). Haematological toxicity was also significantly less common with 5-FU infusion, underlining the improved therapeutic ratio provided by this approach (Meta-analysis Group In Cancer, 1998).

Drawing on the experience gained with 5-FU infusion, two new avenues of drug development have been explored. The direct thymidylate synthase (TS) inhibitors are a group of rationally designed molecules that compete with folinic acid for binding to TS (Figure 1). Intracellular polyglutamation of some of these compounds leads to their retention within the cell, and hence prolonged inhibition of TS. Raltitrexed, the lead agent in this class, has been the subject of extensive clinical evaluation. Its efficacy at the established dose of 3 mg $m^{-2}$ every 3 weeks appears comparable to either bolus or infused regimens of 5-FU/folinic acid (Cunningham et al, 1996; Harper, 1997; Pazdur and Vincent, 1997; Maughan et al, 1999) (Table 1). However, one of the four randomized controlled trials conducted to date demonstrated a significantly inferior median survival with raltitrexed compared with the Mayo regimen of bolus 5-FU and folinic acid given daily × 5 every 4–5 weeks (9.7 vs 12.7 months, $P = 0.011$) (Pazdur and Vincent, 1997). The toxicity profile of raltitrexed is different to that of folinic acid-modulated 5-FU, producing significantly less mucositis and leucopenia, but a greater incidence of elevated transaminases. Of some concern, one study reported a toxic death rate of 5.6% associated with raltitrexed (Maughan et al, 1999). A significant number of these patients had mild–moderate renal impairment, which appears to be an important determinant of severe toxicity with this agent. We would currently recommend assessing renal function prior to administration of raltitrexed, and to proceed only if the creatinine clearance is greater than 65 ml $min^{-1}$.

Other direct TS inhibitors have entered clinical evaluation, but have not as yet been compared with established regimens in randomized trials. One such, nolatrexed is a lipophilic molecule, entering cells by diffusion and lacking a requirement for the reduced folate carrier for transport. It also lacks a glutamate side-chain and is therefore not polyglutamated. Thus it has the potential to overcome some of the resistance mechanisms observed with

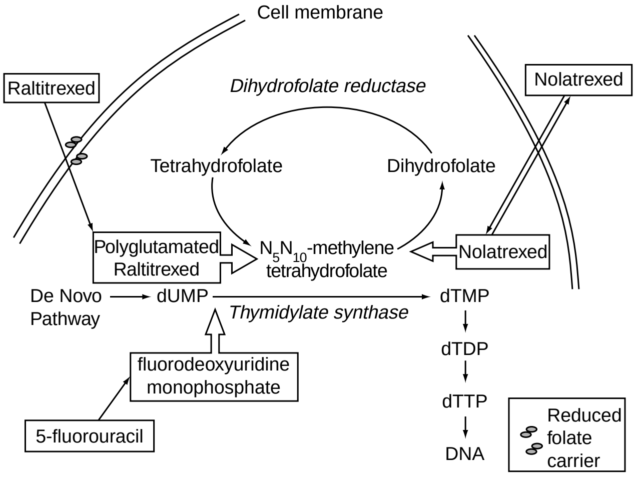

**Figure 1** Direct and indirect thymidylate synthase inhibitors

*Received 31 March 2000*
*Revised 20 September 2000*
*Accepted 6 October 2000*

*Correspondence to:* D Cunningham. E-mail: dcunn@icr.ac.uk

**Table 1** Randomized trials comparing raltitrexed with 5-fluorouracil regimens

| Trial | Cunningham et al, 1996 | | Harper, 1997 | | Pazdur and Vincent, 1997 | | Maughan et al, 1999 | | |
|---|---|---|---|---|---|---|---|---|---|
| Arm | Raltitrexed | 5-FU + LDFA | Raltitrexed | 5-FU + LDFA | Raltitrexed | 5-FU + LDFA | Raltitrexed | Lokich | de Gramont |
| Patients (*n*) | 223 | 216 | 230 | 222 | 199 | 179 | 301 | 301 | 303 |
| Response rate (%) | 19.3 | 16.7 | 18.6 | 18.1 | 14.3 | 15.2 | 20 | 26 | 24 |
| Median overall survival (months) | 10.1 | 10.2 | 10.9 | 12.3 | 9.7 | 12.7 | 10 | 10 | 10 |
| *P* value for survival | 0.42 | | 0.197 | | 0.01 | | Not significant | | |

5-FU + LDFA = Mayo Clinic regimen of bolus 5-FU plus low-dose folinic acid days 1–5 every 4–5 weeks; Lokich = protracted venous infusion 5-FU; de Gramont = folinic acid infusion followed by 5-FU bolus and 22-h infusion on two consecutive days every 2 weeks; Raltitrexed = 3 mg $m^{-2}$ iv bolus every 3 weeks.

many other compounds in this class. Phase I trials have established nausea and vomiting as the dose-limiting toxicities of oral nolatrexed (Hughes et al, 1999; Jodrell et al, 1999), and phase II studies investigating this agent in a number of solid tumours including colorectal cancer are underway.

A second approach to the problem of achieving continuous inhibition of TS without the requirement for protracted intravenous infusion of 5-FU has been the development of orally bioavailable fluoropyrimidines. 5-FU itself has erratic oral bioavailability, precluding oral administration. This problem has been overcome by the use of prodrugs of 5-FU that are well absorbed from the gastrointestinal tract and enzymatically converted to 5-FU in the liver or within the tumour itself. UFT is a combination of the prodrug tegafur and uracil in a 1:4 molar ratio. Uracil is a reversible inhibitor of the enzyme dihydropyrimidine dehydrogenase, which is responsible for the catabolism of 5-FU. UFT 300–350 mg $m^{-2}$ $day^{-1}$ given in combination with oral folinic acid 150 mg $m^{-2}$ $day^{-1}$ for 28 days followed by a 7-day rest produced a response rate of 42% in a phase II study in 45 chemotherapy-naive patients (Pazdur et al, 1994). Grade III diarrhoea was seen in five out of seven patients treated at the higher dose level but only in four of 38 at the reduced dose, which was therefore recommended for further evaluation. This combination has been compared with intravenous bolus administration of 5-FU/folinic acid in two randomized clinical trials involving 1196 patients in total (Table 2). In the North American trial, the standard Mayo Clinic regimen was used as the control arm (Pazdur et al, 1999), whereas in the European trial the modified Mayo regimen was used, in which bolus 5-FU and folinic acid are administered daily for 5 consecutive days every 5 weeks (Carmichael et al, 1999). The response rates to UFT/folinic acid were 12% in these studies, and were statistically equivalent to those produced by the Mayo regimens. The median overall survival duration was also equivalent in the two arms but the North American study showed a significantly poorer median time to disease progression in the UFT/folinic acid arm. Severe toxicity was seen less frequently with the oral formulation, particularly with regard to neutropenia and infection, although hyperbilirubinaemia was more common.

Capecitabine is a fluoropyrimidine carbamate that is activated in three enzymatic steps. The final activating enzyme is the tumour-associated angiogenesis factor thymidine phosphorylase, thus reducing exposure of normal tissues to the active metabolite. In a phase II study comparing three schedules of administration in 109 chemotherapy-naive patients with advanced CRC, response rates of 24% were seen, with one third of patients experiencing grade III toxicity. Median time to progression was 30 weeks with the optimal schedule of 2510 mg $m^{-2}$ $day^{-1}$ given intermittently. Capecitabine has also been evaluated in two randomized, controlled trials (Table 2) (Cox et al, 1999; Twelves et al, 1999). The Mayo regimen was also selected as the control arm in these studies. Tumour response rates were higher in the capecitabine arms in both studies (27% vs 18% and 23% vs 16%, both $P \leq 0.02$), and progression-free survival and overall survival were equivalent between the two arms. The toxicity profile of capecitabine was broadly favourable in comparison with the Mayo regimen, producing less neutropenia and mucositis, although palmar-plantar erythema was more common with the oral compound.

It is clear from the data discussed above that several approaches to TS inhibition have been applied successfully to patients with advanced colorectal cancer. As might be expected, the activity of

**Table 2** Randomized trials comparing two oral fluoropyrimidines with bolus 5-FU/folinic acid

| Trial | Twelves et al, 1999 | | Cox et al, 1999 | | Pazdur et al, 1999 | | Carmichael et al, 1999 | |
|---|---|---|---|---|---|---|---|---|
| Arm | Capecitabine | 5-FU + LDFA[a] | Capecitabine | 5-FU + LDFA[a] | UFT + FA | 5-FU + LDFA[b] | UFT + FA | 5-FU + LDFA[a] |
| Patients (*n*) | 301 | 301 | 302 | 303 | 816 | | 380 | |
| Response rate (%) | 26.6 | 17.9 | 23.2 | 15.5 | 12 | 15 | 11 | 9 |
| *P* value for response | 0.013 | | 0.02 | | 0.232 | | 0.593 | |
| Median PFS (months) | 5.5 | 4.9 | 4.4 | 5.1 | 3.5[c] | 3.8[c] | 3.4[c] | 3.3[c] |
| *P* value for PFS | 0.67 | | Not significant | | 0.011 | | 0.591 | |
| Median overall survival (months) | 13.71 | 3.0 | Not reported | | 12.4 | 13.4 | 12.2 | 11.9 |
| *P* value for survival | 0.68 | | – | | Not significant | | 0.682 | |

Capecitabine = 2500 mg $m^{-2}$ $day^{-1}$ for 2 weeks out of each 3-week cycle; UFT 300 mg $m^{-2}$ $day^{-1}$ and folinic acid 75 or 90 mg $day^{-1}$ for 28 days out of every 35-day cycle; 5-FU + LDFA = Mayo clinic regimen of bolus 5-FU plus low-dose folinic acid days 1–5 every 4[a] or 5[b] weeks; [c]Time to progression quoted

*© 2001 Cancer Research Campaign*

each approach is similar, although differences in toxicity profile and ease of administration have been demonstrated. It is perhaps significant that most of the randomized trials performed with the newer agents have employed the relatively toxic Mayo regimen as the control arm, possibly enhancing the appearance of their tolerability. However, the advantage of being able to dispense with indwelling venous catheters is substantial, and is likely to lead to the gradual replacement of 5-FU as the fluoropyrimidine of choice. The similarity of the mechanism of action of these agents means that activity in 5-FU-resistant patients is likely to be modest at best. This has been borne out in the few studies to address this question to date (Farrugia et al, 1998). Thus the availability of new agents with distinct intracellular targets was required to enhance survival rates for patients with this disease.

### IRINOTECAN

Irinotecan (CPT-11, Campto) is a semi-synthetic camptothecin derivative that inhibits DNA topoisomerase I. It has been shown to have activity against colorectal cancer cell lines in vitro, and proved manageable in phase I clinical trials using a variety of schedules. The most important toxicities associated with its use are diarrhoea, neutropenia, nausea, alopecia, cholinergic syndrome and asthenia. The incidence of severe diarrhoea is significantly reduced by the early and aggressive use of loperamide, and the cholinergic syndrome is prevented in the majority of patients by premedication with anticholinergic agents. The occurrence of severe diarrhoea concomitantly with severe neutropenia is, however, life-threatening, requiring immediate hospital admission and the institution of supportive measures. During phase II development, irinotecan was found to show activity both in chemotherapy-naive and in 5-FU-resistant patients, and was therefore initially formally evaluated in randomized trials as a second-line treatment. In order to quantify the impact of second-line irinotecan therapy on survival and quality of life, a prospective multicentre randomized phase III clinical trial was designed to compare irinotecan with best supportive care in this setting (Cunningham et al, 1998). 279 patients were randomized on a 2:1 basis. Hence 189 patients were randomized to receive irinotecan 350 mg $m^{-2}$ every 3 weeks for up to eight cycles, and 90 patients were randomized to supportive care alone. Patients were required to have documented progression of metastatic colorectal cancer while on 5-FU or within 6 months of the last 5-FU infusion, and to have had no more than two prior 5-FU regimens for metastatic disease. 71% of patients had tumour-related symptoms. With a median follow-up of 13 months, the median overall survival was significantly better in the irinotecan group (9.2 vs 6.5 months, $P = 0.0001$). 1-year survival was 2.6 times greater in the irinotecan group (36.2% irinotecan vs 13.8% supportive care). The survival advantage adjusted for prognostic factors remained highly significant. Grade III–IV toxicity in the irinotecan arm comprised neutropenia in 22% of patients (3% with febrile neutropenia), diarrhoea in 22%, vomiting in 14%, and mucositis in 2% of patients. Severe asthenia and pain occurred more frequently in the supportive care arm. Survival without Performance Status (PS) deterioration, without weight loss >5% and pain-free survival were significantly better in the irinotecan group ($P$ = 0.0001, 0.018 and 0.003, respectively). Global health status assessed by the European Organization for Research and Treatment of Cancer (EORTC) QLQ-C30 quality of life questionnaire was also significantly better in the irinotecan arm ($P < 0.001$), and time to deterioration of quality of life was longer in this group ($P < 0.002$). This study clearly demonstrated the advantage of irinotecan over no specific treatment in good performance status patients following 5-FU failure, both in overall survival and in quality of life.

A second large multicentre phase III trial compared irinotecan with one of three 5-FU infusion regimens (protracted venous infusion 5-FU, weekly high-dose 24-h infusion 5-FU with folinic acid or the de Gramont regimen of bimonthly folinic acid and 5-FU bolus plus 48-h infusion, depending on local practice). Patients had documented progressive disease within 3 months of adjuvant 5-FU or their first palliative 5-FU regimen (Rougier et al, 1998). 157 patients were randomized. 62% of patients had been previously treated with a bolus 5-FU regimen and 38% had received a prior infused 5-FU regimen. 13% had only previously received adjuvant 5-FU. With a median follow-up of 15 months, median survival was significantly longer in the irinotecan arm (10.8 months vs 8.5 months; $P = 0.04$), with a 1-year survival 1.4 times greater than in the 5-FU infusion group (44.8% vs 32.4% respectively). Progression-free survival was also significantly prolonged in the irinotecan group, with a median of 4.2 months compared with 2.9 months in the 5-FU group ($P = 0.029$). Quality of life was maintained in both groups, and toxic effects were similar, although diarrhoea and vomiting were more common in the irinotecan arm. Once again, in multivariate analysis, treatment arm remained a significant favourable prognostic factor for survival. Taken together, these two studies clearly established a role for irinotecan in patients with 5-FU-refractory advanced colorectal cancer who are sufficiently fit for treatment.

Two multicentre randomized controlled trials have evaluated the addition of irinotecan to standard bolus or infused regimens of 5-FU and folinic acid as first-line therapy for advanced colorectal cancer (Table 3). The results of these studies formed the basis of a submission to the Oncologic Drugs Advisory Committee of the Food and Drug Administration (FDA) of the USA, which approved irinotecan for the first-line treatment of advanced colorectal cancer. The first study (Douillard et al, 2000), conducted in Europe and South Africa, evaluated the addition of irinotecan to one of two infused 5-FU/folinic acid regimens: the de Gramont regimen, or a weekly regimen of folinic acid and 24-h 5-FU infusion (the regimen to be used was selected prior to commencement of the study by each participating centre). 385 patients were randomized in the study. There was a statistically significant difference in objective response rate (34.8% vs 21.9%, $P = 0.005$), overall survival (17.4 vs 14.1 months, $P = 0.031$), and time to progression (6.7 vs 4.4 months, $P < 0.001$) in favour of the irinotecan-containing arm. Toxicity was increased in patients treated with irinotecan. Diarrhoea, neutropenia and asthenia were increased in frequency and severity compared with patients receiving 5-FU/folinic acid alone, and one toxic death occurred in the irinotecan group as a result of septic shock and diarrhoea. However, quality of life scores were similar in the two arms, and time to deterioration of quality of life was longer in the irinotecan arm. The second study was conducted in the USA and involved 683 patients (Saltz et al, 1999, 2000). Randomization was between three treatment arms: the Mayo regimen of bolus 5-FU/folinic acid; weekly irinotecan alone; weekly irinotecan plus bolus 5-FU and folinic acid for 4 of every 6 weeks. However the primary analysis compared only the irinotecan/5-FU/folinic acid and 5-FU/folinic acid arms. An updated analysis of this trial presented at the annual meeting of the American Society of Clinical Oncology in May 2000 has shown improved response rates (39.4% vs 20.8%, $P < 0.0001$), time to progression (7.0 vs 4.3 months, $P = 0.004$) and overall survival (14.8 vs 12.6 months,

© 2001 Cancer Research Campaign

**Table 3** Randomized trials of combination chemotherapy in the first-line treatment of advanced colorectal cancer

| Trial | Treatment arm | Patients (*n*) | Response rate (%) | *P* value | Median TTP (months) | *P* value | Median OS (months) | *P* value |
|---|---|---|---|---|---|---|---|---|
| V307 Douillard et al, 2000 | Irinotecan + 5-FU/FA[a] | 198 | 35 | <0.005 | 6.7 | 0.001 | 17.4 | 0.032 |
| | 5-FU/FA[a] | 187 | 22 | | 4.4 | | 14.1 | |
| 0038 Saltz et al, 1999 | Irinotecan + 5-FU/FA[b] | 231 | 39 | <0.001 | 7.0 | 0.004 | 14.8 | 0.042 |
| | 5-FU/FA[b] | 226 | 21 | | 4.3 | | 12.6 | |
| | Irinotecan | 226 | 18 | – | 4.2 | – | 12.0 | – |
| 2961 Giacchetti et al, 1997 | Oxaliplatin + 5-FU/FA[c] | 100 | 34 | 0.001 | 8.3 | 0.0003 | 17.4 | 0.58 |
| | 5-FU/FA[c] | 100 | 12 | | 4.2 | | 19.2 | |
| 2962 de Gramont et al, 2000 | Oxaliplatin + 5-FU/FA[a] | 210 | 49 | 0.001 | 9.0 | 0.0003 | 16.2 | 0.12 |
| | 5-FU/FA[a] | 210 | 22 | | 6.2 | | 14.7 | |
| Ross et al, 1997 | Mitomycin C + PVI 5-FU | 98 | 54 | 0.024 | 7.9 | 0.033 | 14.0 | ns |
| | PVI 5-FU | 97 | 38 | | 5.4 | | 12.7 | |

[a]de Gramont regimen of 5-FU and folinic acid; [b]Modified Mayo regimen; [c]Chronomodulated infusion of 5-FU and folinic acid. See text for details of regimens.

*P* = 0.042) associated with the combination treatment. The combination of 5-FU and irinotecan produced more grade III or IV diarrhoea, asthenia and alopecia than the Mayo schedule. However, it was associated with a similar incidence of severe neutropenia and fewer episodes of febrile neutropenia and mucositis. Toxic death rates were less than 2%, and were similar in the three arms. This study also confirmed that quality of life was similar between treatment arms. A combined analysis of the results from these two trials showed hazard ratios for time to disease progression of 0.67 ($P < 0.001$) and survival of 0.79 ($P < 0.009$) in favour of the combination treatment (Saltz et al, 2000).

### OXALIPLATIN

Oxaliplatin is a third-generation platinum complex with single-agent activity in colorectal cancer. Importantly, synergy is demonstrated between oxaliplatin and 5-FU both in vitro and in chemotherapy-naive patients. There have been no randomized trials of oxaliplatin-containing regimens as second-line therapy in 5-FU-resistant colorectal cancer reported to date. However, a number of phase II trials have demonstrated the feasibility of combining oxaliplatin with various 5-FU/folinic acid regimens in this setting, including the modified Mayo regimen (Van Cutsem et al, 1999) bimonthly 48-h infusion regimens (de Gramont et al, 1997; Maindrault-Goebel et al, 1998,1999), and weekly high-dose 24-h infusion (Janinis et al, 1999; Van Cutsem et al, 1999). Response rates have ranged from 11–46%, with median survival up to 17 months, suggesting that such combinations may have a useful role. The contribution of 5-FU to the efficacy of these regimens in patients with 5-FU-resistant disease has been called into question. Some of the more recent trials have demonstrated response rates similar to those produced by single-agent oxaliplatin (Van Cutsem et al, 1999).

Oxaliplatin plus 5-FU/folinic acid has been compared with 5-FU/folinic acid alone as first-line treatment for advanced colorectal cancer in two large randomized phase III trials (Table 3). 420 patients were randomized to receive a regimen of folinic acid and 5-FU bolus plus 24-h infusion on two consecutive days every 2 weeks plus or minus oxaliplatin 85 mg $m^{-2}$ every 2 weeks (de Gramont et al, 2000). Median progression-free survival was superior in the oxaliplatin arm (9 months vs 6.2 months, *P* = 0.0003). Median overall survival also showed a trend to superiority in the oxaliplatin arm (16.2 months vs 14.7 months, *P* = 0.12). Cross-over from the 5-FU/folinic acid arm to the oxaliplatin/5-FU/folinic acid arm following progression may account for the smaller difference in median survival seen between the two arms. Furthermore, when subjected to multivariate analysis, randomization to the oxaliplatin-containing arm was a statistically significant favourable prognostic factor for survival. Quality of life was also evaluated in this study. Patients randomized to receive oxaliplatin had a significantly prolonged survival without 20% (*P* = 0.0039), or 40% (*P* = 0.0004) deterioration in quality of life. In the second trial, 200 patients received the same chronomodulated 5-day infusion of 5-FU (700 mg $m^{-2}$ $day^{-1}$) plus folinic acid (300 mg $m^{-2}$ $day^{-1}$) with or without oxaliplatin (125 mg $m^{-2}$ day 1), with courses repeated every 3 weeks. The median progression-free survival was 7.9 months in the oxaliplatin arm, and 4.3 months in the control arm (*P* = 0.05). Median overall survival showed no significant difference between arms (17.6 months oxaliplatin, 19.4 months control, *P* = 0.82) (Giacchetti et al, 1997). An analysis of the use of second-line chemotherapy and surgery for metastases in the two arms of this trial showed that both of these treatment modalities were more commonly employed in the control arm, and were likely to account for the lack of a significant survival benefit in the oxaliplatin arm (Giacchetti et al, 1998).

### COMBINATIONS OF IRINOTECAN AND OXALIPLATIN

The differing mechanism of action and non-overlapping dose-limiting toxicity profiles of these two agents provides a good

 © 2001 Cancer Research Campaign rationale for their use in combination. This has been investigated in a number of phase I/II trials from which some preliminary results are available. The combination appears manageable and effective in patients with 5-FU-refractory disease using a variety of schedules including weekly (Kemeny et al, 2000), 2-weekly (Goldwasser et al, 1999; Wasserman et al, 1999) and 3-weekly dosing (Wasserman et al, 1999). Reported response rates have ranged from 17–64% in this patient population.

### MITOMYCIN C

Mitomycin C has been in use for the treatment of metastatic colorectal cancer for many years with response rates of 0–30%. In a multicentre randomized trial including 200 patients with chemotherapy-naive disease, the addition of mitomycin C to a protracted venous infusion of 5-FU improved response rates (54% vs 38% $P$ = 0.024), and median failure-free survival (7.9 vs 5.4 months, $P$ = 0.033) compared with protracted venous infusion 5-FU alone (Table 3) (Ross et al, 1997). However, there were no overall survival differences between the two arms. The combination produced higher rates of haematological toxicity, and particularly thrombocytopenia, but non-haematological toxicity was similar in the two arms. Haemolytic uraemic syndrome was seen in two patients treated at the initial mitomycin C dose of 10 mg mg$^{-2}$ every 6 weeks, but in no patients treated at a reduced dose of 7 mg m$^{-2}$.

### CONCLUSIONS

The debate on the management of advanced colorectal cancer has moved on from the question of whether to use palliative chemotherapy to the question of which drugs to use, and in what sequence. A major issue for current debate is whether to use drugs in sequence or in combination. As yet no completed clinical trials have addressed this question fully. However, a number of clues are available from the studies outlined above. Firstly, it is clear that patients who are fit enough to receive second-line chemotherapy after 5-FU failure will derive benefit from it. This has been demonstrated unequivocally in the case of irinotecan, with two randomized clinical trials providing supportive data. It is also likely to be true for oxaliplatin, although formal randomized trials testing this hypothesis have not been reported as yet. It is much less probable that the oral fluoropyrimidines or the direct thymidylate synthase inhibitors will provide significant benefits in this setting, although different mechanisms of resistance to some of these agents may allow scope for their use in this patient population.

A potential drawback of the sequential approach is that a proportion of patients will not be fit enough for second-line chemotherapy after 5-FU failure due to rapid disease progression, and may therefore be denied exposure to an agent to which their disease may be sensitive. The use of combination chemotherapy as first-line treatment may overcome this problem. This is suggested by the results of the two trials comparing irinotecan plus 5-FU/folinic acid with 5-FU/folinic acid alone. Despite the fact that 31% and 50% of patients in the latter arm of the two trials went on to receive second-line irinotecan, a survival benefit for the combination chemotherapy was observed (Saltz et al, 1999; Douillard et al, 2000). The lack of a similar survival advantage in the two trials evaluating oxaliplatin plus 5-FU/folinic acid in the first-line setting may reflect the better performance status of the patients enrolled in these trials, leading to a greater proportion of patients being suitable for second-line chemotherapy. Randomized trials formally comparing different treatment algorithms in which first-, second- and even third-line treatment choices are stipulated by the trial protocol may be required to determine the optimal approach. Combination chemotherapy unquestionably increases response rates, which may have an important bearing on patients with limited metastatic disease that might be amenable to surgical resection.

Although currently only the subject of speculation, it may be anticipated that improvements in response rates to neoadjuvant chemotherapy may result in increased rates of long-term survival following metastasectomy. For this reason complete response rates to chemotherapy are becoming a more important end-point in evaluating the efficacy of new combination regimens. At the present time, the data supporting the use of a combination of 5-FU and irinotecan in the first-line treatment of colorectal cancer is difficult to resist. Such a combination approach is rapidly becoming standard across North America for patients treated outside clinical trials. Toxicity is increased over infused 5-FU regimens, but is similar to that produced by the Mayo Clinic regimen of bolus 5-FU and folinic acid, and does not impair quality of life. It is difficult to explain the differing overall survival outcomes of the oxaliplatin and irinotecan first-line combination studies in view of the very similar response rates and time to tumour progression produced by either drug in combination with 5-FU/folinic acid. A trial reported in preliminary form earlier this year investigates the efficacy and toxicity of a combination of irinotecan, 5-FU and folinic acid followed after disease progression by a combination of oxaliplatin, 5-FU and folinic acid. This is compared in a randomized fashion with the same two combination regimens given in the reverse sequence (Tournigand et al, 2000). The response rates to the first regimen in the two arms are very high, and are equivalent (63% for the irinotecan combination and 60% for the oxaliplatin combination). This trial may provide evidence for the optimal order in which to give the available active drugs. Until such evidence is available, other factors such as the acceptability to the patient of the toxicity profile of the drug, its convenience of administration and its cost are important considerations (Table 4).

The oral fluoropyrimidines and direct thymidylate synthase inhibitors provide a substantial advance in the convenience of chemotherapy administration. Preliminary data suggest that some of these drugs can be administered safely in combination with either irinotecan or oxaliplatin, and result in promising activity.

**Table 4** Comparison of oxaliplatin and irinotecan

| Oxaliplatin | Irinotecan |
|---|---|
| **Toxicity** | |
| This drug has a well documented cumulative and only partially reversible neurotoxicity | As far as we are aware, no irreversible toxicity |
| Diarrhoea and neutropenia occur but are less common than with irinotecan | Diarrhoea and neutropenia occur more commonly, but patient education and adherence to protocol lowers risk of severe toxicity |
| No alopecia | Alopecia common, but less frequent with 2-weekly schedules |
| **Pharmacy considerations** | |
| Administered as a 2-h infusion | Administered as a 30-minute infusion |
| Requires reconstitution prior to use | No requirement for reconstitution |
| **Cost** | |
| The cost of these drugs appears roughly equivalent | |

Combinations of oxaliplatin and irinotecan are also being evaluated and are the subject of an ongoing randomized North American trial. Three drug regimens incorporating a fluoropyrimidine, oxaliplatin and irinotecan may also be feasible, and have the potential to elevate response rates even further, although toxicity is likely to be increased. Regimens in which irinotecan and oxaliplatin, each in combination with a fluoropyrimidine, are alternated from cycle to cycle may overcome some of the toxicity problems of the three-drug combination. This may also delay the onset of oxaliplatin-induced neurotoxicity, which is related to the cumulative exposure to this drug. An alternative approach is to tailor the treatment regimen to the disease using molecular markers that might predict sensitivity to individual agents. There is excellent data to suggest that high thymidylate synthase expression within the tumour predicts for poor response to 5-FU (Leichman et al, 1997; Salonga et al, 2000) or raltitrexed (Ford et al, 2000), and topoisomerase I is being explored as a marker for sensitivity to irinotecan. A caveat to this approach is that expression of these enzymes can vary substantially between the primary tumour and metastatic disease within the same patient. It follows that analysis of biopsies of the metastatic tumour deposits is necessary to apply this technology in a rational manner. A synthesis of these data is presented in Figure 2 in the form of an algorithm for the treatment of advanced colorectal cancer patients.

The extensive experience that has been gained with the new agents discussed above in the treatment of advanced colorectal cancer is now being brought forward to the adjuvant setting. It is likely that eradication of micrometastatic disease will be maximized by using chemotherapy regimens with very high response rates in patients with advanced disease. Standard 5-FU/folinic acid regimens are being compared with combination regimens incorporating oxaliplatin or irinotecan plus 5-FU/folinic acid in ongoing randomized trials in Europe and North America to test this hypothesis, although the advances seen with combination chemotherapy in metastatic disease are likely to be reflected in the adjuvant setting.

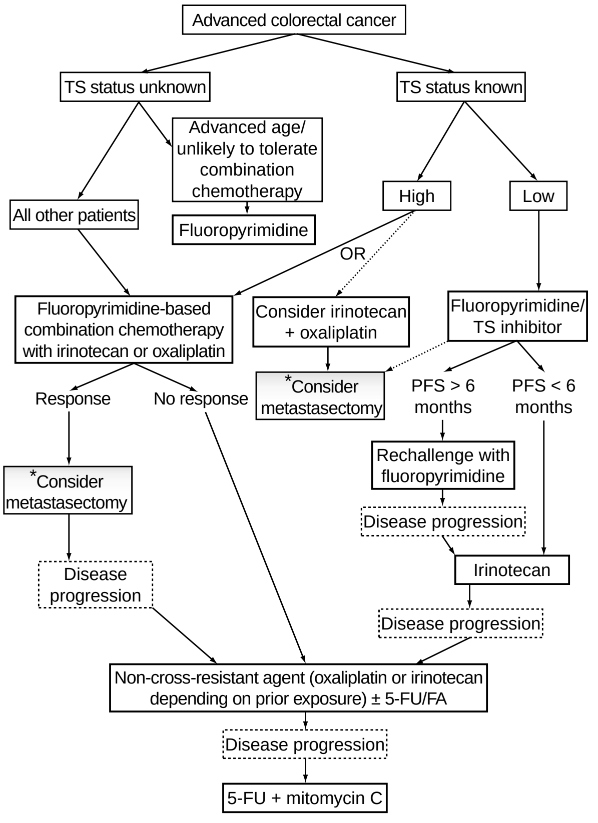

**Figure 2** An algorithm for the treatment of advanced colorectal cancer (metastasectomy should be considered for patients with metastatic disease confined to liver or lung)

### REFERENCES

Advanced Colorectal Cancer Meta-Analysis Project (1992) Modulation of fluorouracil by leucovorin in patients with advanced colorectal cancer: evidence in terms of response rate. *J Clin Oncol* **10**: 896–903

Carmichael J, Popiela T, Radstone D, Falk S, Fey M, Oza A, Skovsgaard T and Martin C (1999) Randomized comparative study of ORZEL (oral uracil/tegafur (UFT) plus leucovorin) versus parenteral 5-fluorouracil plus leucovorin in patients with metastatic colorectal cancer (abstract). *Proc Am Soc Clin Oncol* **18**: 264a

Cox JV, Pazdur R, Thibault A, Maroun J, Weaver C, Jahn MW, Harrison E and Griffin T (1999) A phase III trial (SO 14695) of Xeloda (capecitabine) in previously untreated advanced/metastatic colorectal cancer (abstract). *Proc Am Soc Clin Oncol* **18**: 265a

Cunningham D, Zalcberg JR, Rath U, Oliver I, van Cutsem E, Svensson C, Seitz JF, Harper P, Kerr D and Perez Manga G (1996) Final results of a randomised trial comparing 'Tomudex' (raltitrexed) with 5-fluorouracil plus leucovorin in advanced colorectal cancer. 'Tomudex' Colorectal Cancer Study Group. *Ann Oncol* **7**: 961–965

Cunningham D, Pyrhonen S, James RD, Punt CJA, Hickish TF, Heikkila R, Johannesen T, Starkhammar H, Topham CA, Awad L, Jacques C and Herait P (1998) Randomised trial of irinotecan plus supportive care versus supportive care alone after fluorouracil failure for patients with metastatic colorectal cancer. *Lancet* **352**: 1413–1418

de Gramont A, Vignoud J, Tournigand C, Louvet C, Andre T, Varette C, Raymond E, Moreau S, Le Bail N and Krulik M (1997) Oxaliplatin with high-dose leucovorin and 5-fluorouracil 48-hour continuous infusion in pretreated metastatic colorectal cancer. *Eur J Cancer* **33**: 214–219

de Gramont A, Figer A, Seymour M, Homerin M, Hmissi A, Cassidy J, Boni C, Cortes-Funes H, Cervantes A, Freyer G, Papamichael D, Le Bail N, Louvet C, Hendler D, de Braud F, Wilson C, Morvan F and Bonetti A (2000) Leucovorin and fluorouracil with or without oxaliplatin as first-line treatment in advanced colorectal cancer. *J Clin Oncol* **18**: 2938–2947

Douillard JY, Cunningham D, Roth AD, Navarro M, James RD, Karasek P, Jandik P, Iveson T, Carmichael J, Alakl M, Gruia G, Awad L and Rougier P (2000) Irinotecan combined with fluorouracil compared with fluorouracil alone as first-line treatment for metastatic colorectal cancer: a multicentre randomised trial. *Lancet* **355**: 1041–1047

Farrugia DC, Norman AR and Cunningham D (1998) Single agent infusional 5-fluorouracil is not effective second-line therapy after raltitrexed (Tomudex) in advanced colorectal cancer. *Eur J Cancer* **34**: 987–991

Ford HER, Farrugia DC, Cunningham D, Aherne GW, Hardcastle A, Mitchell F, Hill ME, McCarthy K, McVicar D, Danenberg PV, Danenberg K and Jackman AL (2000) High thymidylate synthase mRNA expression may predict non-response in tumor and increased risk of toxicity in normal gastrointestinal mucosa following treatment with raltitrexed ('Tomudex') (abstract). *Proc Am Soc Clin Oncol* **19**: 243a

Giacchetti S, Zidani R and Perpoint B (1997) Phase III trial of 5-fluorouracil, folinic acid, with or without oxaliplatin in previously untreated patients with metastatic colorectal cancer (abstract). *Proc Am Soc Clin Oncol* **16**: 229a

Giacchetti S, Brienza S, Focan C, Metouri A, Perpoint B, Faggiuolo R, Llory JF, Le Rol A, Larregain-Fournier D, Tigaud JM, Debotte G, Itzhaki M, Misset JL and Levi F (1998) Contribution of second line oxaliplatin-chronomodulated 5-fluorouracil-folinic acid and surgery to survival in metastatic colorectal cancer patients (abstract). *Proc Am Soc Clin Oncol* **17**: 273a

Goldwasser F, Gross M, Tigaud JM, Jasmin C, Marceau-Suissa J, Misset JL and Cvitkovic E (1999) CPT-11/Oxaliplatin combination every two weeks: Final results of a phase I study in advanced digestive malignancies (abstract). *Proc Am Soc Clin Oncol* **18**: 176a

Harper P (1997) Advanced colorectal cancer: results from the last (raltitrexed) Tomudex comparative study (abstract). *Proc Am Soc Clin Oncol* **16**: 228a

Hughes AN, Rafi I, Griffin MJ, Calvert AH, Newell DR, Calvete JA, Johnston A, Clendeninn N and Boddy AV (1999) Phase I studies with the nonclassical antifolate nolatrexed dihydrochloride (AG337, THYMITAQ) administered orally for 5 days. *Clin Cancer Res* **5**: 111–118

© 2001 Cancer Research Campaign

Janinis J, Fountzilas G, Skarlos DV, Efstathiou E, Aravantinos G and Papakostas P (1999) Second-line combination chemotherapy with weekly oxaliplatin and high dose 5-fluorouracil with leucovorin in metastatic colorectal cancer. A Hellenic Cooperative Oncology Group study (abstract). *Proc Am Soc Clin Oncol* **18**: 255a

Jodrell DI, Bowman A, Rye R, Byrne B, Boddy A, Rafi I, Taylor GA, Johnston A and Clendeninn NJ (1999) A phase I study of the lipophilic thymidylate synthase inhibitor Thymitaq (nolatrexed dihydrochloride) given by 10-day oral administration. *Br J Cancer* **79**: 915–920

Kemeny N, Tong W, Stockman J, Blanchette J and Saltz L (2000) Phase I trial of weekly oxaliplatin and irinotecan in previously treated patients with metastatic colorectal cancer (abstract). *Proc Am Soc Clin Oncol* **19**: 245a

Leichman CG, Lenz HJ, Leichman L, Danenberg K, Baranda J, Groshen S, Boswell W, Metzger R, Tan M and Danenberg PV (1997) Quantitation of intratumoral thymidylate synthase expression predicts for disseminated colorectal cancer response and resistance to protracted-infusion fluorouracil and weekly leucovorin. *J Clin Oncol* **15**: 3223–3229

Maindrault-Goebel F, De Gramont A, Louvet C, Andre T, Carola E, Gilles-Amar V, Izrael V, Molitor JL and Krulik M (1998) Bi-monthly oxaliplatin with leucovorin and 5-fluorouracil in pretreated metastatic colorectal cancer (FOLFOX 6) (abstract). *Proc Am Soc Clin Oncol* **17**: 273a

Maindrault-Goebel F, De Gramont A, Louvet C, Andre T, Carola E, Gilles-Amar V, Lotz JP, Izrael V and Krulik M (1999) High-dose oxaliplatin with the simplified 48 h bimonthly leucovorin and 5-fluorouracil regimen in pretreated metastatic colorectal cancer (abstract). *Proc Am Soc Clin Oncol* **18**: 265a

Maughan TS, James RD, Kerr D, McArdle C, Ledermann JA, Seymour M, Johnston C and Stephens RJ (1999) Preliminary results of a multicentre randomised trial comparing three chemotherapy regimens (de Gramont, Lokich and raltitrexed) in metastatic colorectal cancer (abstract). *Proc Am Soc Clin Oncol* **18**: 262a

Meta-analysis Group In Cancer (1998) Efficacy of intravenous continuous infusion of fluorouracil compared with bolus administration in advanced colorectal cancer. *J Clin Oncol* **16**: 301–308

Pazdur R and Vincent M (1997) Raltitrexed (Tomudex) vs 5-fluorouracil + leucovorin in patients with advanced colorectal cancer: results of a randomised multicentre North American trial (abstract). *Proc Am Soc Clin Oncol* **16**: 228a

Pazdur R, Lassere Y, Rhodes V, Ajani JA, Sugarman SM, Patt YZ Jones DV, Jr, Markowitz AB, Abbruzzese JL, Bready B and Levin B (1994) Phase II trial of uracil and tegafur plus oral leucovorin: an effective oral regimen in the treatment of metastatic colorectal carcinoma. *J Clin Oncol* **12**: 2296–2300

Pazdur R, Douillard J-Y, Skillings JR, Eisenberg PD, Davidson N, Harper P, Vincent MD, Lembersky BC and Benner SE (1999) Multicentre phase III study of UFT in conbination with leucovorin in patients with metastatic colorectal cancer (abstract). *Proc Am Soc Clin Oncol* **18**: 263a

Ross P, Norman A, Cunningham D, Webb A, Iveson T, Padhani A, Prendiville J, Watson M, Massey A, Popescu R and Oates J (1997) A prospective randomised trial of protracted venous infusion 5-fluorouracil with or without mitomycin C in advanced colorectal cancer. *Ann Oncol* **8**: 995–1001

Rougier P, Van Cutsem E, Bajetta E, Niederle N, Possinger K, Labianca R, Navarro M, Morant R, Bleiberg H, Wils J, Awad L, Herait P and Jacques C (1998) Randomised trial of irinotecan versus fluorouracil by continuous infusion after fluorouracil failure in patients with metastatic colorectal cancer. *Lancet* **352**: 1407–1412

Salonga D, Danenberg KD, Johnson M, Metzger R, Groshen S, Tsao-Wei DD, Lenz HJ, Leichman CG, Leichman L, Diasio RB and Danenberg PV (2000) Colorectal tumors responding to 5-fluorouracil have low gene expression levels of dihydropyrimidine dehydrogenase, thymidylate synthase, and thymidine phosphorylase. *Clin Cancer Res* **6**: 1322–1327

Saltz LB, Locker PK, Pirotta N, Elfring GL and Miller LL (1999) Weekly irinotecan, leucovorin, and fluorouracil is superior to daily × 5 LV/FU in patients with previously untreated metastatic colorectal cancer (abstract). *Proc Am Soc Clin Oncol* **18**: 233a

Saltz LB, Douillard J, Pirotta N, Awad L, Elfring GL, Gruia G, Locker PK, Alakl M, Knight RD and Miller LL (2000) Combined analysis of two phase III randomised trials comparing irinotecan, fluorouracil, leucovorin versus fluorouracil alone as first-line therapy of previously untreated metastatic colorectal cancer (abstract). *Proc Am Soc Clin Oncol* **19**: 242a

Tournigand C, Louvet C, André T, Achille E, Lledo G, Flesch M, Ganem G, Landi B, Risse M-L, Lotz V, Colin P and de Gramont A (2000) FOLFIRI followed by FOLFOX or FOLFOX followed by FOLFIRI in metastatic colorectal cancer: Which is the best sequence? Safety and preliminary efficacy results of a randomized phase III study (abstract). *Proc Am Soc Clin Oncol* **19**: 245a

Twelves C, Harper P, Van Cutsem E, Thibault A, Shelygin YA, Burger HU, Allman D and Osterwalder B (1999) A phase III trial (SO 14796) of Xeloda (capecitabine) in previously untreated advanced/metastatic colorectal cancer (abstract). *Proc Am Soc Clin Oncol* **18**: 263a

Van Cutsem E, Szanto J, Roth A, Humblet Y, Köhne CH, Wils J, Lorenz M, Borner M, Schöffski P and Tabah-Fisch I (1999) Evaluation of the addition of oxaliplatin to the same Mayo or German 5FU regimen in advanced refractory colorectal cancer (abstract). *Proc Am Soc Clin Oncol* **18**: 234a

Wasserman E, Kalla S, Misset JL, Goldwasser F, Bedairia N, Bensamine MA, Marty M and Cvitkovic E (1999) Oxaliplatin and irinotecan phase I/II studies: Results in 5-FU refractory colorectal cancer patients (abstract). *Proc Am Soc Clin Oncol* **18**: 238a

*© 2001 Cancer Research Campaign*
